# The effect of the inner engineering program on interpersonal relationships: a qualitative study of emotional regulation and relational transformation

**DOI:** 10.3389/fpsyg.2025.1595878

**Published:** 2025-10-31

**Authors:** Enas Mohamed, Arlene Katz, Nashaw Jafari, Akila Rayapuraju, Preeti Upadhyay Reed, Sepideh Hariri, Balachundhar Subramaniam

**Affiliations:** ^1^Sadhguru Center for a Conscious Planet, Department of Anesthesia, Critical Care & Pain Medicine, Beth Israel Deaconess Medical Center, Boston, MA, United States; ^2^Harvard Medical School, Boston, MA, United States

**Keywords:** inner engineering meditation, Shambhavi Mahamudra Kriya, relationships, emotional regulation, transformation

## Abstract

**Introduction:**

Relationship difficulties are a major source of anxiety in contemporary society, contributing to social withdrawal, emotional reactivity, and conflict, which in turn increase isolation and distress. Meditation has emerged as a promising strategy for managing these stressors by fostering self-awareness, resilience, and empathy.

**Methods:**

This study explores the Inner Engineering (IE) program, a structured, self-paced meditation intervention developed by the Isha Foundation. The program consists of seven 90-minute modules incorporating guided meditation, reflective writing, and practices such as Shambhavi Mahamudra Kriya (SMK), which integrates alternate nostril breathing, AUM chanting, breath awareness, and postural yoga.

**Results:**

Findings suggest that meditation practices included in IE support emotional regulation, cognitive reappraisal, and the cultivation of positive affect, contributing to healthier interpersonal relationships. Theories of mindfulness and self-compassion further indicate that such structured training can enhance adaptability, self-acceptance, and interpersonal stability.

**Discussion:**

The IE program highlights meditation’s role as a non-religious, scalable intervention for improving well-being in the context of relational stress. By combining ancient yogic practices with modern reflective exercises, IE may provide individuals with effective tools to manage emotional reactivity, foster empathy, and strengthen relationships.

## Introduction

Relationship issues, especially in today’s fast-paced and stressful environments, are significant sources of anxiety. These challenges often lead to social withdrawal, heightened emotional reactivity, and difficulties in conflict management, which can cause strain in relationships with partners, friends, and family members to deteriorate, increasing isolation and emotional distress (Kasalova et al., 2018). Meditation and mindfulness have emerged as effective interventions for managing these relationship-related stressors. By engaging in mindful breathing, emotional regulation, and cognitive reappraisal, individuals can enhance self-awareness, emotional resilience, and empathy—key components for maintaining positive, balanced relationships (Upadhyay et al., 2022a,b).

Psychological theories on self-compassion (Neff, 2003) and mindfulness (Counselling Collective, 2024) suggest that structured meditation practices cultivate inner stability, self-acceptance, and adaptability, enabling individuals to respond skillfully to interpersonal stressors. Similarly, attachment theory (Bowlby, 1982; Mikulincer and Shaver, 2007; Cassidy et al., 2013; Flaherty and Sadler, 2011) and emotion regulation models (Gross, 1998, 2015) emphasize how such practices can support secure relationships and adaptive emotional responses. These perspectives guided our analysis of participants’ experiences with SMK.

The IE program, offered by the Isha Foundation, is a meditation course designed to enhance overall well-being and quality of life (Upadhyay et al., 2022b). In the IE program, participants learn SMK, which incorporates alternate nostril breathing, AUM chanting, and breath awareness (Upadhyay et al., 2022b). Developed by Jagadish Vasudev (Sadhguru), this program emphasizes cognitive reappraisal, positive emotion generation, guided meditation, and inner energy activation through sound and postural yoga (Vasudev, 2016). The IE program is a self-paced, non-religious online intervention consisting of seven 90-min modules, with reflective writing exercises after each module to deepen participants’ awareness and integrate insights into daily life (Upadhyay et al., 2022a). In the present study, however, data were derived from semi-structured interviews rather than the written reflections.

Numerous quantitative studies have examined the effects of IE on well-being, stress, and other psychological factors (Upadhyay et al., 2022a; Upadhyay et al., 2022b). Research has shown that IE enhances resilience, mindfulness, and subjective well-being, providing valuable insights into its benefits. However, existing literature has lacked a qualitative analysis of IE’s impact on interpersonal relationships. This study addresses the lack of qualitative research on IE by exploring its impact on relational dynamics and self-perception. Through in-depth, semi-structured interviews, we examined the psychological mechanisms and transformative processes by which IE fosters change in relationships and self-awareness. These insights contribute valuable context for integrating contemplative practices within therapeutic and relational frameworks, broadening the applications of IE in mental health and interpersonal domains.

## Methods

### Study design

We conducted a qualitative study using conventional content analysis (Hsieh and Shannon, 2005) in semi-structured individual interviews to understand the effect of IE on participants’ interpersonal relationships. The semi-structured interviews allowed us to gain rich, descriptive information about their overall experience of the practice (Hutcherson et al., 2008), as well as the various effects of IE on their interpersonal skills.

### Sampling and recruitment

The Isha Foundation facilitated participant recruitment for the study. We used purposive sampling to invite individuals who had completed the IE program, were 18–75 years old, and resided in the United States, with all participants providing consent to share their contact information. To ensure variation, recruitment included individuals from multiple geographic locations.

A total of 24 participants (17 females, 7 males; mean age = 56 years) were enrolled. The sample was 71% female and predominantly in their fifties, reflecting self-selection among those who chose to participate rather than intentional targeting. This trend aligns with broader patterns in contemplative practice, where middle-aged and older adults are more likely to engage, possibly due to greater life experience, interest in meaning and growth, or more flexibility in time and resources (Cramer et al., 2016).

Recruitment occurred among individuals who had voluntarily enrolled in the IE program, often after exposure to Sadhguru’s teachings through YouTube, books, or introductory meditation practices. As this was a large-scale, fee-based online program from Isha Foundation, promoted globally to millions, our sample represents individuals who self-selected into IE and consented to participate. For most, IE was their initial entry point into meditation with Isha Foundation, and they had no prior experience before registering. We acknowledge that this recruitment pathway may have introduced selection bias, as participants may have been predisposed to valuing IE. While this limits generalizability, the study’s goal was to capture participants’ lived experiences of completing the program rather than represent attitudes in the general population. To mitigate bias, we used purposive sampling to maximize variation in demographics (e.g., gender, age, geography) and designed semi-structured interviews to allow participants to reflect on their experiences openly.

### Ethics approval and consent

This study was approved by the Beth Israel Deaconess Medical Center Review Board (protocol: IRB-2022P000587). All participants were electronically asked to give their informed consent. Once we obtained electronically signed consent forms, we allowed a six-week period to pass after the completion of the IE program before commencing the qualitative interviews.

### Data collection

Single, semi-structured interviews were conducted with 17 female and 7 male participants, six weeks after completing the IE program. The interview guide focused on two topics: (1) participants’ experiences with SMK and (2) their self-perceptions before and after six weeks of practice. Although the IE program also included reflective writing exercises, only interview data were analyzed for this study.

All interviews were conducted remotely via Microsoft Teams to enable participation across different U. S. states. They were held in English, lasted 60–90 min, and were video- and audio-recorded with consent. Variability in interview length reflected participants’ communication style and level of detail shared, but both shorter and longer interviews yielded rich data that contributed to theme development. The full interview guide is available in Supplementary Appendix 1.

### Data analysis and rigor (following COREQ guidelines)

Data were analyzed using an inductive, conventional thematic approach, guided by COREQ standards for rigor and transparency (Hsieh and Shannon, 2005; Knafl, 1991). Interviews were transcribed and checked against audio recordings for accuracy. A subset of transcripts was open-coded to identify emergent themes, which were labeled, described, and supported with quotes. Two team members reviewed the codebook, and discrepancies were resolved through discussion. The finalized codebook was then applied to the whole dataset using Dedoose (v9.0.107). Three analysts examined the coded data to derive initial themes representing participants’ experiences with SMK.

Although coding was primarily inductive, interpretation was informed by psychological theories on self-compassion (Neff, 2003), mindfulness (Kabat-Zinn, 1990), attachment (Bowlby, 1982; Mikulincer and Shaver, 2007), and emotion regulation (Gross, 1998, 2015). To ensure rigor, multiple analysts independently coded subsets of transcripts, compared results, and resolved differences through consensus. An audit trail documenting coding decision, codebook iterations, and theme refinement was maintained. Weekly team meetings supported theme review and refinement. Thematic saturation was achieved after approximately the 20th interview, with the final four confirming no new codes or themes.

The analysis yielded a final set of three descriptive categories and themes (A-C below).

### Reflexivity

The analytic team approached the data primarily as outsiders to the IE program. The majority of researchers had no direct experience with IE or SMK, and their knowledge was limited to publicly available information. This outsider stance supported a focus on participants’ lived experiences as described in the interviews, rather than on insider assumptions. To minimize interpretive bias, team members engaged in ongoing discussions about how their perspectives might influence coding and theme development. In addition, the semi-structured interview guide was designed to elicit open, in-depth reflections, ensuring that findings were grounded in participants’ own words rather than researchers’ preconceptions.

## Results

The sample included 24 participants: females had a mean age of 56 years and males a mean age of 43 years. Participants represented a range of ethnic and educational backgrounds, though the majority identified as Caucasian. Demographic characteristics are summarized in Table 1.

**Table 1:** Sociodemographic characteristics of the study participants by gender.

Characteristics	Overall *n* (%)	Female *n* (%)	Male *n* (%)
Age group			
18–30	3 (12.5)	2 (8.3)	1 (4.1)
31–40	6 (25)	4 (16)	2 (8)
41–50	5 (20)	3 (12.5)	2 (8)
>50	10 (41)	8 (33)	2 (8)
Age (mean & range)		56 (53)	43 (25)
Number of kids (mean & range)		1 (5)	2 (4)
Education level			
High School	4 (16)	1 (4.1)	3 (12.5)
College	5 (20)	4 (16)	1 (4.1)
Bachelor’s	6 (25)	6 (25)	0 (0)
Masters	7 (29)	3 (12.5)	2 (8.3)
PhD	2 (8.3)	2 (8.3)	0 (0)
MD	2 (8.3)	1 (4.1)	1 (4.1)
Ethnicity			
Caucasian	14 (58)	11 (45)	2 (8.3)
Indian	7 (29)	4 (16)	3 (12.5)
Hispanic	1 (4.1)	0 (0)	1 (4.1)
Non-Hispanic	1 (4.1)	1 (4.1)	0 (0)
Chinese	1 (4.1)	1 (4.1)	0 (0)
Religion			
Christianity	17 (70)	13 (54)	4 (16)
Hindu	7 (29)	4 (16)	3 (12.5)
Immigrants	10 (41)	6 (25)	4 (16)

### Overview

All participants who regularly practiced IE for 21 min once per day for six weeks reported noticeable changes in their interpersonal relationships, though the nature and extent of these changes varied. Analysis revealed three overarching domains of change: (A) Emotional Healing and Regulation, (B) Cultivating Self-Compassion and Empathy, and (C) Relationship Harmony and Resilience. Within these domains, participants described experiences such as releasing longstanding pain, adapting to life transitions, deepening kindness toward self and others, and improving communication and conflict resolution. Each domain is elaborated through subthemes and illustrated with participant narratives below.

#### Emotional healing and regulation

##### Emotional resilience

Study members talked about how practicing SMK helped them find inner peace and heal from pain. They reported finding that their body and mind are more aligned, and having a more profound sense of connection to something very significant, which resulted in healing and a sense of tranquility.

*“I have let those hurt feelings and pain go, and I can be nice to myself and laugh now. I let go of the pain feelings caused by my dad, I feel stronger, my mind and body are aligned, and this alignment makes me sense a connection with the divine”* (71y, F, PhD, Caucasian)

##### Adaptation and restoration

Several immigrant Interviewees who left their home countries and moved to the USA reported a significant improvement in their mental health and emotional well-being after practicing.

SMK. Many shared that before adopting the practice, they often felt easily upset, depressed, and anxious, particularly due to the challenges of immigration and the added stress of the COVID-19 pandemic. However, through SMK, participants described a sense of returning to their “old selves” while also attaining a “better state” of overall well-being.

*“After moving to the USA and leaving behind all the people I love in Albania, especially during the isolation of COVID, I faced significant challenges. I became depressed, anxious, and emotionally dysregulated. I used to get easily upset and cry, and I started seeing a therapist. Then, through social media, I learned about the inner engineering program, and after trying it, it brought me home. Shambhavi brought me back to my old self and brought me to a better state, where you can notice a change in the mind and emotions, if that makes sense.”* (39y, F, College, non-Hispanic)

“*I have been able to handle changes in my life much more easily. Since practicing Shambhavi, I feel less stressed and anxious about major life events, such as moving to the USA, living alone in an apartment, and starting a new job. In the past, these changes would overwhelm me, often leading to feelings of defeat and tears. Adjusting to a new culture and meeting new people was also difficult, adding to the sense of isolation and stress. Now, I find it easier to approach these situations with a calm mindset, embracing new experiences rather than reacting emotionally as I used to*.*”* (24y, F, College, Indian)

##### Transforming suffering

With the practice of SMK, many reported that they felt a sense of relief from the sadness they had been carrying. This sadness was often due to the loss of a family member or difficult childhood experiences with their parents. Additionally, a few participants mentioned that doing SMK helped them build better relationships with people who reminded them of those who had caused them pain in the past.

*“My boyfriend and I used to be lovers, and I got to where I could barely stand to be around him. Because he reminds me of my dad, and all those emotions come up while being around him. I feel hurt when he is around me, despite being nice to me. I was so mean to him, and of course, I realized that wasn’t about him. After I learned Shambhavi, I learned lots of tools, like how to let go of emotions. And I was able to let go of all that pain about my dad, and now I’m not mean to him anymore.”* (71Y, F, PhD, Caucasian)

*“My father killed himself when I was 12 and I definitely had a very hard time forgiving him, even with it being like so many years later, but after doing Shambhavi, I just see it in a whole different way now, and I’m kind of more okay with it now.”* (32y, F, College, Caucasian)

#### Cultivating self-compassion and empathy

##### Developing kindness toward self and others

Several participants shared their profound experiences of kindness and love that stemmed from practicing SMK. They noted that these feelings were not temporary but persisted after the practice, having a long-lasting and significant effect on their lives.

*“Shambhavi brings emotion that’s really kind of hard to describe, you can say it brings moments of kindness and an intense joy, that lasts even after you finish the practice.”* (42y, M, High School African American)

##### External validation of inner change

Voices in the interviews conveyed that members of their immediate circle have noticed positive changes in them. For example, their partners noticed that they have become happier since starting SMK and less anxious about work or financial issues.

*“I do not know if I recall my wife’s exact words, but she said something like you just seem to be a little happier or easier to get along with, also you stopped getting anxious about work and financial issues.”* (55Y, M, Bachelor, Caucasian)

In addition, participants’ coworkers noticed that the participants were calmer and more composed than they were before learning SMK.

*“My coworkers have noticed a change in me, as one of my friends told me, “I can see that you are becoming less anxious and calmer.”* (42 Y, F, PhD, Indian)

#### Relationship harmony and resilience

##### Increased communication engagement

Some reflected that they used to struggle with patience in their interactions with others, such as patients visiting their clinics or with their children. However, after attending the IE program, participants in medical professions reported that they did not struggle to connect with their patients.

Furthermore, parents have also started putting in effort to communicate effectively with their children. In general, participants noticed improved communication with others through engaging in more active listening and checking in with others more regularly.

*“I think an awareness of being much more present when my patients talk to me. Some people they are talking too much, or what they are talking about does not interest me, and I’m trying to bear with it. But I reach a point where I feel so uninterested in listening, and it’s so boring to talk. Now after the Shambhavi, I’m connecting to that person’s heart; I’m seeing them as, ok, they need to share for whatever reason, even if it’s not interesting to me. But as a human connection, I’m here for them.”* (53yo, F, MD, France)

*“Before Shambhavi, I would not put in so much effort to communicate with my kids. I’d call If they do not respond, I would not call back. I will say he does not want to call or whatever he’s busy with. But now I put in a little more effort to communicate with my kids; we have just waited for them to ask for help in solving their problems. But now, I try and reach out to them more, even if they are not responding.”* (55yo, M, Bachelor, Indian)

##### Enhancing understanding and respect for autonomy

Narratives highlighted changes in participants’ interactions with their children following the practice of SMKIn the past, they would meddle with their children’s decisions or yell at them to get their attention which caused a rift between them and their children. Practicing SMK enabled them to become less agitated and reduce reactive outbursts. Participants show signs of wanting to engage in more open communication with their children. They began to understand their children’s demands without shouting or getting anxious.

*“I have not been able to get my son to pay attention to a normal or firm speaking voice. I was yelling on him because he just wasn’t responding, and he used to get upset. But now the yelling is less, and I’m trying now to figure out what he needs without yelling at him”* (34yo, F, Bachelor, Caucasian)

Experiences revealed a transition from a controlling, anxiety-driven approach to one defined by acceptance and trust in their adult children’s autonomy. They also demonstrated greater acceptance of their adult children’s decisions and a heightened respect for their boundaries.

*“Before I used to interfere a lot with my son life, and he used to get mad and stops answering my phone calls, and that makes me so worry. But now it’s like, ok, it’s his life. He′ll figure it out. he is mature enough, I started to be calmer, less anxious and give him a space to think and chose what he wants.”* (73y, F, High School, Caucasian)

##### Improved conflict resolution with partners

Many reported that they had fewer arguments with their partners, as they could control their nerves and not react immediately. When practicing SMK, participants demonstrated de-escalation techniques, including learning to respond rather than react, taking breaks to revisit situations when calmer and more collected, and accepting their partner.

*“Before, when my husband does something or said something makes me angry, I used to yell, shout, slam doors, or stomp off, as a way to show him I’m mad. Now I think about my response before I say it. I think, “What am I thinking right now? Oh, I’m feeling really angry. I cannot believe that he would make that suggestion.” That’s my first thought. Then my next thought is. “Oh, I’m feeling really hurt,” and then my third thought is, “I think I’m going to hold off on seeing anything because I do not want to hurt him, and I do not want to make it worse right now*.” (59, F, College, Caucasian)

*“I stopped arguing with my husband on small things; in general, we have poor communication with each other, and sometimes we argue about small things for the sake of arguing; there is a cultural difference between us, and we can argue on simple things. For example, I say tomato, and he says tommatto, so we start arguing about the correct pronunciation, so after Shambhavi, I stopped arguing with him on small things, and started to accept the difference between us.”* (40, F, Bachelor, Indian).

## Discussion

Although no studies have directly examined the neural mechanisms of SMK, related research provides plausible pathways that may underlie the benefits reported by participants. Neuroimaging evidence shows that mindfulness meditation reduces amygdala reactivity to emotional stimuli and strengthens connectivity with prefrontal regions involved in emotion regulation (Kral et al., 2018). Similarly, fMRI studies of “OM” chanting demonstrate deactivation of limbic structures, including the amygdala, suggesting a calming effect on stress-related circuits (Kalyani et al., 2011). More recently, a pilot study on unilateral nostril breathing—an element of SMK—found that such practices may enhance psychological well-being and cognitive functioning by modulating autonomic balance (Vanutelli et al., 2024). Taken together, these findings suggest that SMK’s integration of mindfulness, chanting, and breath regulation may plausibly reduce amygdala hyperreactivity, enhance prefrontal–amygdala coupling, and shift autonomic activity toward parasympathetic dominance, thereby supporting the emotional regulation and resilience observed in our study.

Consistent with these mechanisms, participants reported notable gains in emotional control, empathy, and self-compassion. This aligns with prior evidence that meditation fosters positivity, reduces negative emotions, and enhances empathy and social connectedness (Hutcherson et al., 2008). Specifically, loving-kindness meditation has been recognized as a powerful tool for improving relationship quality, with studies showing that even when only one partner practices, the overall health of the relationship may improve (Hutcherson et al., 2008; Somohano, 2013). Such practices bring awareness, presence, and nonjudgmental attention to interpersonal interactions, supporting active listening and thoughtful responses over reactivity. Our qualitative findings complement and extend quantitative evidence on IE. For example, Upadhyay et al. (2022a) and Upadhyay et al. (2022b) found that IE Online reduced stress and enhanced joy and mindfulness among IT professionals, with the most substantial benefits observed among those who practiced daily. Similarly, improvements in resilience, mindfulness, and subjective well-being have been reported (Upadhyay et al., 2022b). While these studies demonstrate measurable outcomes, our analysis highlights the experiential processes underlying such change, including emotional regulation, transformation of suffering, and improved relationship dynamics—mechanisms not captured by quantitative metrics alone.

Participants’ narratives consistently emphasized personal empowerment and transformation in their relationships. They described enhanced mindfulness, acceptance, and communication skills that supported conflict resolution and respect for personal boundaries. Gratitude and self-awareness were frequently reported, with positive feedback from others reinforcing these changes. For many Caucasian participants, IE acted as a catalyst for emotional healing, alleviating distress and fostering inner peace. Empowered self-love emerged as a cornerstone, enabling participants to release past trauma and cultivate resilience.

Among immigrant participants, meditation supported adaptation to cultural transitions often accompanied by stress, anxiety, and depression. Prior research has documented meditation’s role in fostering resilience during life changes (Liu et al., 2022), and our findings are consistent with this. Immigrant participants described how IE provided inner stability and alignment of mind, body, and spirit, which promoted belonging, reduced reactivity, and improved confidence in communication. The program also helped them rediscover joy and cultivate a more positive outlook, facilitating smoother cultural integration.

These findings carry significant clinical implications. SMK may be considered a complementary approach in therapeutic contexts to support emotional regulation, resilience, and relational wellbeing (Goodman and Sommers-Flanagan, 2019). Clinicians may integrate SMK as an adjunct to existing interventions for stress, anxiety, and trauma recovery, particularly for patients seeking contemplative or non-pharmacological options. Significantly, physicians themselves may benefit from practicing SMK as a means of managing occupational stress. While further controlled trials are needed to establish efficacy and inform clinical guidelines, the present findings suggest that SMK can enrich integrative care models by providing accessible tools to cultivate calm, self-awareness, and healthier interpersonal relationships.

### Strengths and limitations

We are aware of several limitations in our study. Firstly, the sample was not randomly selected; 71% of participants were women, and the mean age was 56. The absence of younger adults and relatively few male participants may influence the findings, particularly as prior research suggests potential gendered differences in emotional regulation and responsiveness to contemplative practices (e.g., women often report greater emotional expressivity, while men may engage in different regulatory strategies; Chaplin et al., 2019). Similarly, younger adults may face distinct developmental needs and stressors compared to middle-aged participants, which were not fully captured here. The relatively small sample size (24 participants) also limits the extent to which these findings can be applied more broadly.

Nevertheless, an essential strength of the study is that it captured the effects of regular SMK practice across diverse educational backgrounds, ethnicities, and age ranges among both women and men. To our knowledge, this represents the first qualitative study exploring the impact of the IE program—and specifically SMK—on interpersonal relationships. Future research with larger, more diverse, and balanced samples will be essential to further explore potential differences across gender and age groups and to validate these preliminary insights.

## Conclusion

The IE program and SMK serve as promising, transformative tools for enhancing interpersonal relationships and emotional well-being. Within six weeks of daily practice, participants reported noticeable improvements in communication, relationship quality, conflict resolution, and overall resilience. These findings suggest that SMK may help individuals cultivate emotional regulation, empathy, and self-compassion, which extend to more harmonious interactions with partners, children, and colleagues. While the study provides important insights, limitations such as the modest sample size, gender and age imbalance, and reliance on self-selected participants should be considered when interpreting results. Nevertheless, the findings carry potential clinical relevance: SMK could be explored as a complementary practice in therapeutic and wellness contexts to support individuals dealing with stress, trauma, or relational challenges. Future research should employ larger and more diverse samples, as well as mechanistic and longitudinal designs, to deepen understanding of how SMK influences psychological and relational outcomes.

## Data Availability

The data analyzed during the current study are available from the corresponding author on reasonable request. Access is subject to institutional and ethical approval requirements.
