# Supplementary material for: The effect of the inner engineering program on interpersonal relationships: a qualitative study of emotional regulation and relational transformation
Source: Front Psychol. 2025 Oct 31;16:1595878. doi: 10.3389/fpsyg.2025.1595878 (PMC12615163; doi:10.3389/fpsyg.2025.1595878)
Supplement: Supplementary file 1 [file Supplementary_file_1.pdf]

Qualitative Interview Guide: Participant Experiences with Shambhavi Meditation (IECO Program)

| Main Question | Example Probes |
|---|---|
| Can you tell me about your experience with meditation before joining this program? | Had you practiced meditation before, or was this your first time?<br>- If it was your first time, what motivated you to participate?<br>- What had you heard about the program that influenced your decision? |
| If you had practiced before, please share more about those experiences. | - Have you known or spoken to anyone who practices meditation? What impressions did you get from them?<br>- If you haven't, what do you imagine people might say about their reasons for practicing? |
| What made you want to participate in the Shambhavi meditation practice? Why is this important to you? | |
| Would you please walk me through your experience of the Shambhavi initiation process? | - Think of a moment that stayed with you during or after initiation. |
| Can you describe how things feel different after six weeks of practice compared to the very beginning? | - What changes have you noticed in your body, mind, or emotions?<br>- Any changes in behaviors, feelings, or habits?<br>- Did you expect any changes when you began? |
| How would you describe yourself before the Shambhavi practice? How do you view yourself now? | |
| | |

| | |
|---|---|
| How would you describe your interaction with people before and after the IECO program? | - Who do you interact with daily, and who are you closest to?<br>- Has the quality of your relationships changed?<br>- Have others noticed any changes in you? |
| How did you approach life challenges before and after the IECO program? | - Have your thoughts or reactions changed?<br>- Tell me about a recent challenge and how you handled it. |
| After completing six weeks of Shambhavi practice, are there any experiences or insights that have stayed with you? | |
| What advice would you give to people who want to try the Shambhavi practice? | |
| Are you planning to continue with the practice? | - If yes, why?<br>- If no, why not? |
